# A rapid, non-invasive, clinical surveillance for CachExia, sarcopenia, portal hypertension, and hepatocellular carcinoma in end-stage liver disease: the ACCESS-ESLD study protocol

**DOI:** 10.1186/s12876-023-03093-8

**Published:** 2023-12-21

**Authors:** Patrik Nasr, Mikael Forsgren, Wile Balkhed, Cecilia Jönsson, Nils Dahlström, Christian Simonsson, Shan Cai, Anna Cederborg, Martin Henriksson, Henrik Stjernman, Martin Rejler, Daniel Sjögren, Gunnar Cedersund, Wolf Bartholomä, Ingvar Rydén, Peter Lundberg, Stergios Kechagias, Olof Dahlqvist Leinhard, Mattias Ekstedt

**Affiliations:** 1 Department of Health, Medicine and Caring Sciences, Linköping University, Linköping, Sweden; 2 Wallenberg Centre for Molecular Medicine, Linköping University, Linköping, Sweden; 3 Center for Medical Image Science and Visualization (CMIV), Linköping University, Linköping, Sweden; 4 Department of Internal Medicine, Ryhov Hospital Jönköping, Jönköping, Sweden; 5 Department of Medicine, Höglandssjukhuset Eksjö, Region Jönköping County Council, Jönköping, Sweden; 6 The Jönköping Academy for Improvement of Health and Welfare, Hälsohögskolan, Jönköping University, Jönköping, Sweden; 7 Department of Biomedical Engineering, Linköping University, Linköping, Sweden; 8 School of Medical Sciences and Inflammatory Response and Infection Susceptibility Centre (iRiSC), Faculty of Medicine and Health, Örebro University, Örebro, Sweden; 9 Department of Research, Region Kalmar County, Kalmar, Sweden; 10 Department of Biomedical and Clinical Sciences, Linköping University, Linköping, Sweden; 11 AMRA Medical AB, Linköping, Sweden; 12 Institute of Medicine, Sahlgrenska Academy, University of Gothenburg, Gothenburg, Sweden; 13 Department of Medicine, Sahlgrenska University Hospital, Gothenburg, Sweden

**Keywords:** Liver cirrhosis, Hepatocellular carcinoma, Portal hypertension, Sarcopenia, Biomarkers, Abbreviated MRI

## Abstract

**Background:**

Liver cirrhosis, the advanced stage of many chronic liver diseases, is associated with escalated risks of liver-related complications like decompensation and hepatocellular carcinoma (HCC). Morbidity and mortality in cirrhosis patients are linked to portal hypertension, sarcopenia, and hepatocellular carcinoma. Although conventional cirrhosis management centered on treating complications, contemporary approaches prioritize preemptive measures. This study aims to formulate novel blood- and imaging-centric methodologies for monitoring liver cirrhosis patients.

**Methods:**

In this prospective study, 150 liver cirrhosis patients will be enrolled from three Swedish liver clinics. Their conditions will be assessed through extensive blood-based markers and magnetic resonance imaging (MRI). The MRI protocol encompasses body composition profile with Muscle Assement Score, portal flow assessment, magnet resonance elastography, and a abbreviated MRI for HCC screening. Evaluation of lifestyle, muscular strength, physical performance, body composition, and quality of life will be conducted. Additionally, DNA, serum, and plasma biobanking will facilitate future investigations.

**Discussion:**

The anticipated outcomes involve the identification and validation of non-invasive blood- and imaging-oriented biomarkers, enhancing the care paradigm for liver cirrhosis patients. Notably, the temporal evolution of these biomarkers will be crucial for understanding dynamic changes.

**Trial registration:**

Clinicaltrials.gov, registration identifier NCT05502198. Registered on 16 August 2022. Link: https://classic.clinicaltrials.gov/ct2/show/NCT05502198.

## Background

Chronic liver diseases affects approximately 1 billion individuals worldwide and causes an estimated 2 million deaths annually [1]. The most common causes of liver-related mortality are chronic hepatitis B, chronic hepatitis C, alcohol-related liver disease and non-alcoholic fatty liver disease (NAFLD) [2, 3]. The end stage of all chronic liver diseases is liver cirrhosis, which entails an increased risk of liver-related complications such as decompensation and hepatocellular carcinoma (HCC).

The transition from chronic liver disease to cirrhosis involves inflammation, fibrogenesis and vascular occlusion causing microvascular changes with sinusoidal remodelling, formation of intrahepatic shunts, and hepatic endothelial dysfunction [4]. This causes increased hepatic resistance and splanchnic vasodilatation which precipitates and exaggerates portal hypertension – the underlying pathophysiological cause of liver decompensation.

The gold standard for estimating and diagnosing portal pressure is hepatic venous pressure gradient (HVPG) – an invasive examination. However, clinical, radiological, and endoscopic signs of decompensation is more commonly used to estimate the severity of the portal pressure – which is highly associated with mortality (ranging from 1 to 60% depending on decompensating events). However, recent studies have shown that non-invasive evaluation of portal pressure and flow with magnetic resonance (MR) imaging (MRI) highly correlates with HVPG, and 7-year follow-up of spleen volume (using CT) have shown strong links to decompensation and HCC occurrence [5, 6].

Beyond the lethality of decompensation, liver cirrhosis also entails an increased risk of developing HCC [7]. If diagnosed early, when the tumor is still small, most patients can receive curative treatment. Therefore, current guidelines recommend screening for HCC biannually with ultrasonography, computed tomography or MRI, where the most common used modality is ultrasonography [8]. However, recent studies have found MRI to have a higher HCC detection rate compared to ultrasound [9], with abbreviated MRI (aMRI) showing high sensitivity for detecting HCC irrespective of protocol or any contrast agent [10].

Sarcopenia, a multifactorial muscle disease typically defined as loss of skeletal muscle mass and function, has gained increased attention in the field of liver cirrhosis during the last decade. In chronic disease, sarcopenia is a progressive disorder associated with adverse outcomes (e.g. falls, fractures and physical disability) and increased mortality and morbidity, especially in elderly patients [11]. In patients with cirrhosis, sarcopenia is present in approximately 40–60%, and associated with increased morbidity and mortality, increased ICU length of stay and poorer treatment results, recognizing sarcopenia as a prognostic risk factor in cirrhosis [11–13]. Recent clinical guidance recommends systematic assessment of sarcopenia in patients with cirrhosis, both for liver transplant decision-making as well as monitoring in those with decompensated cirrhosis undergoing management for sarcopenia [14]. Magnetic resonance imaging is considered the gold standard for body composition assessment, and methods for objective measurement of muscle health unbiased by BMI has been developed, such as the Muscle Assessment Score (MASs) [15]. The components of MAsS, i.e.*,* thigh fat-free muscle volume z-score (FFMVz) and thigh muscle fat index (sex-adjusted MFI), have been shown to be an independent predictor of all-cause mortality and identifies vulnerable patients with fatty liver disease [16, 17].

The field of liver cirrhosis is shifting from being reactive (i.e., treating decompensation once it occurs) to being proactive (i.e.*,* preventing decompensation from happening). For this shift to be successful, we need validated biomarkers that predict prognosis and is reflective of change in disease activity. The aim of the current study is to test blood and imaging-based biomarkers to improve outcome for patients with liver cirrhosis by detecting those patients at risk of developing HCC, sarcopenia, and decompensating events.

Therefore, in this prospective cohort study we aim to recruit patients with cirrhosis undergoing HCC-screening, utilising MRI instead of ultrasonography for repeated imaging every 6 months for 2 years, to investigate the development of HCC, liver stiffness, and development of portal hypertension as wells as body composition profiling including MAsS for detecting sarcopenia. We will assess these aspects with a single, short, clinically feasible, 20 min MR-examination, together with extensive blood sampling for building a biobank for future analyses.

## Methods/design

### Overview

ACCESS-ESLD (A Rapid, Non-invasive, Clinical Surveillance for CachExia, Sarcopenia, Portal HypertenSion, and Hepatocellular Carcinoma in End-Stage Liver Disease) is a multi-center prospective cohort study aiming to include 150 patients with liver cirrhosis. Patients with established liver cirrhosis will be included and repeated abbreviated MR-examinations will be performed instead of ultrasonography at baseline, 6 months, 12 months, and 18 months, i.e., with a 6-month interval. This interval is currently recommended and used in normal clinical care for surveillance of HCC in these patients. The MR-examination will include body composition profiling (BCP; for e.g.*,* sarcopenia [via MAsS] and portal hypertension [via spleen volume]) and HCC-screening. Patients will be consecutively enrolled at the department of Gastroenterology and Hepatology, University Hospital in Linköping, County Hospital in Jönköping (Ryhov) and District Hospital in Eksjö (Höglandssjukhuset). A detailed medical history as well as a comprehensive list of clinical data, biochemical investigation, tests of physical function and mobility, and biological samples will be obtained in all individuals.

### Objectives

The purpose is to identify risk factors as well as biomarkers of present disease severity and future risk of liver-related clinical events in patients with liver cirrhosis. We aim to do this by characterizing body composition profiling, portal blood flow, liver stiffness, and spleen volume.

#### Primary aim

To determine if cross-sectional or temporal changes in FFMV and MFI (i.e.*,* MAsS), in combination or as individual factors, predict the development of ESLD-related events (i.e., decompensation, HCC and sarcopenia) or worsening of liver function (i.e.*,* Model for End-Stage Liver Disease [MELD] score and Child-Turcotte-Pugh score [CTP]).

#### Secondary aims

1. To determine prognostic factors associated with increased mortality.
2. To evaluate non-invasive markers for development of portal hypertension.
3. The diagnostic performance for spleen-based assessment of clinically significant portal hypertension (CSPH) with MRI in predicting events of hepatic decompensation.
4. To investigate the relationship between muscle composition, development of CSPH with health-related quality of life.
5. If the diagnostic performance/robustness of the body composition profile-based assessments improves in combination with:
  i) data driven analysis of population data,
  ii) combinations of/other BCP biomarkers,
  iii) other relevant measurements such as blood samples or genetic composition
  iv) the inclusion of multiple muscle groups in BCP analysis which allows for correcting for confounding factors such as nerve injuries.

### Organization and oversight

The study is run and coordinated by Dr. Mattias Ekstedt (PI) at the Department of Gastroenterology and Hepatology, University Hospital in Linköping, and Faculty of Medicine and Health Sciences, Linköping University. Patient recruitment will take place at the Department of Gastroenterology and Hepatology, University Hospital in Linköping as well as collaborating hospitals; District Hospital in Eksjö and County Hospital in Jönköping, overseen by gastroenterologists participating in the study (local PI for Eksjö and Jönköping are MR and HS, respectively). MRI will be performed at each recruitment site and imaging analysis will be performed by AMRA Medical AB where the coordinators and investigators are MF and ODL. The study will be overseen by Forum Östergötland, which is part of Clinical Studies Sweden.

### Ethical considerations

All recruitment and attainment of informed consent are conducted according to nationally accepted practice and in full accordance with the World Medical Association of Helsinki 2018. Data is collected and processed in accordance with the applicable General Data Protection Regulation (EU) 2016/679 (GDPR) legislation, and in compliance with the International Conference of Harmonization – Good Clinical Practice (ICH-GCP) requirements [18].

The ACCESS-ESLD study was approved by the Swedish Ethical Review Authority 2020–07215, 2021-02-23 with amendments 2022–02902-02 and 2022–06142-02 and is registered as a clinical trial (clinicaltrials.gov identifier NCT 05502198).

### Participants

Patient recruitment will take place at the Gastroenterology and Hepatology clinics at the University Hospital in Linköping, District Hospital in Eksjö and County Hospital in Jönköping. Patients with a clinical diagnosis of cirrhosis are eligible for inclusion and invited to participate by their hepatology nurse or hepatologist during clinical visits. Adult patients of both sexes will be consecutively included. The inclusion and exclusion criteria are presented in Table 1.

**Table 1:** Inclusion and Exclusion criteria

Criteria	
Inclusion	Established liver cirrhosis according to clinical practice criteria Age ≥ 18 years Signed informed consent
Exclusion	Contraindications to perform MRI (pacemaker, ferrous metal implants/fragments, claustrophobia, extreme obesity, and/or pregnancy) Diagnosis of primary sclerosing cholangitis Diagnosis of hepatocellular carcinoma Previous liver transplant

After receiving information about the study and the opportunity to ask questions, participants will be asked to provide written informed consent, witnessed, and dated, by a member of the clinical research team. Written informed consent will always be obtained prior to study-specific procedures.

### Estimation of sample size and power calculation

One of the main objectives of the study concerns sarcopenia in patients with cirrhosis, and sarcopenia is therefore the basis for our power calculations. Previous imaging-based studies on muscle mass and composition in patients with cirrhosis have found sarcopenia to be prevalent in a range of 30 to 70% of patients [19].

At the time of the study design, there were limited numbers of studies with longitudinal follow-up of muscle loss in a somewhat comparable way. One of these studies has shown that an annual loss of muscle volume of − 3.1% (measured by computer tomography) is significantly associated with a high risk of mortality in this patient group [20]. We have previously shown that we can measure muscle volume with a precision of 0.8–1.5% (coefficient of variation) [21, 22].

To detect a change of − 3.1% in a year with a precision of 1.5% (assuming upper limit for safety) and a risk of incorrectly rejecting our hypothesis (α) of 5% and a statistical power (1-β) of 90%, would require a population size of 47. We should reach this population size, with margin, with a total population of 150 people, if about 60% will suffer from muscle loss and we take heed for loss due to e.g.*,* technical errors, study persons who choose to refrain from continued participation, and other unpredictable events that may affect data collection and its quality. A frequently used statistical power of 80% requires 35 people, and we can then assume that if our population is in the low 30% that will suffer from muscle loss, we can still expect to see differences/effects.

### Study procedure

Following the provision of informed consent, patients will be assigned a unique study-participate identification code incorporating the recruitment site identifier. All data will be link-anonymized throughout the study, recorded through a secure web-based application for electronic data (REDCap™, https://projectredcap.org/software/).

A member of the research team will complete a clinical report form on clinical data (Table 2). Questionnaires regarding lifestyle and self-reported quality of life will be obtained, fitness and physical activity will be assessed, and baseline clinical biochemistry will be obtained.

**Table 2:** Layout of the anthropometric and clinical data collected at inclusion and at each visit^a^

Categories of Data	
**Basic data**	
• Date of birth	
• Gender	
• Anthropometrics	
◦ Height	
◦ Weight	
◦ Waist circumference	
◦ Hip circumference	
◦ Blood pressure	
**Previous and present cirrhosis history and severity**	
• Date and modality of cirrhosis diagnosis	
• Underlying etiology	
• Decompensating event (type and date)	
**Assessment of hepatic encephalopathy**	
• Animal naming test	
**Medical History**	
• Previous and present relevant comorbidities and date of diagnosis, including:	
◦ Hypertension, dyslipidemia	
◦ Cardiovascular diseases, including PCI and CABG	
◦ Congestive heart failure	
◦ Atrial fibrillation/flutter	
◦ Stroke	
◦ Malignancies	
• Current or recent medication (including over-the-counter, traditional/herbal remedies, and nutritional supplements)	
**Family History**	
• Family medical history	
**Lifestyle**	
• Smoking – Yes/No/Ex, and frequency of smoking (pack-years)	
• Coffee consumption – cups/days	
• Alcohol consumption	
• Physical activity and fitness	
• Patient reported quality of life	
• Sleep quality assessment	
**Hand grip strength**	
**FibroScan**	
• VCTE	
• CAP	
**MR-examination (20 min)**	
• Body composition: VAT, ASAT, liver PDFF, thigh FFMV and MFI, spinal erector FFMV and MFI, liver volume, and spleen volume.	
• Muscle Assessment Score (MAsS): MVZ and sex-adjusted MFI	
• L3-SMI	
• Liver MRE	
• HCC screening	
**Muscle function and physical frailty**	
• Short physical performance battery (SPPB)	
• Hand grip strength (HGS)	
• Liver frailty index (LFI)	

^a^Follow-up occurs every 6 months, in total 4 MR-examinations. If a lesion defined as LI-RADS-3 is discovered, the interval for surveillance with MRI is shortened to every 4 months for 12 months, in total 5 MR-examinations. If HCC is discovered, study participants’ continuation in the study is seized

*Abbreviations:* *CABG* coronary artery bypass graft, *CAP* controlled attenuation parameter, *FFMV* fat-free muscle volume, *L3-SMI* skeletal muscle index at the 3rd lumbar vertebrae, *MFI* muscle fat infiltration, *MRE* magnetic resonance elastography, *MVZ* muscle volume z-score, *PCI* percutaneous coronary intervention, *PDFF* proton density fat fraction, *ASAT* abdominal subcutaneous adipose tissue, *VAT* visceral adipose tissue, *VCTE* vibration controlled transient elastography

**Fig. 1:**
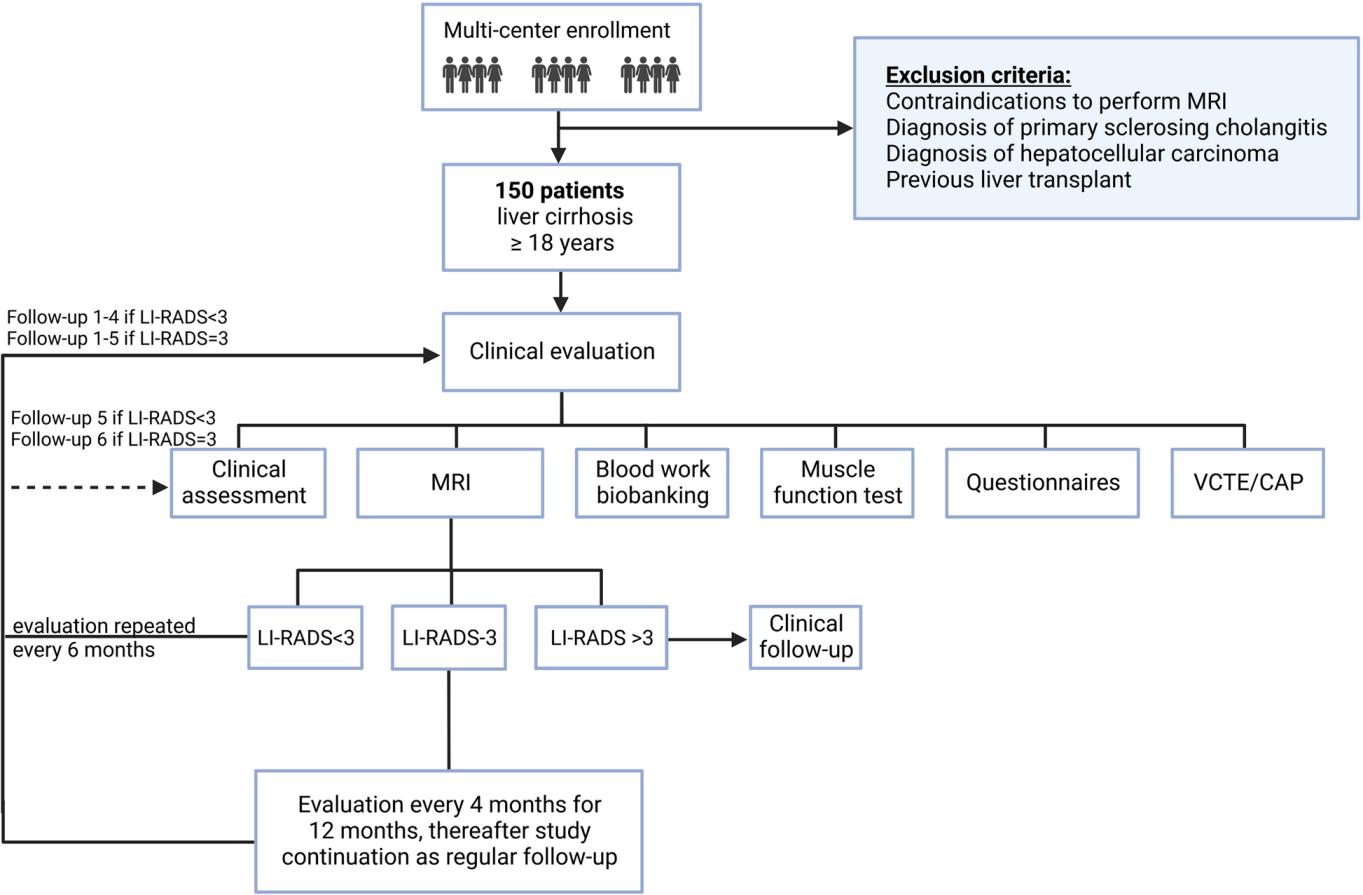
Study flow chart outlining patient recruitment, inclusion, data collection and follow-up. Created with BioRender.com

Included patients are invited for a study visit at the Department of Gastroenterology and Hepatology, University Hospital in Linköping, County Hospital in Jönköping or District Hospital in Eksjö (Fig. 1). The baseline visit is comprised of:

- A clinical visit with a hepatologist or hepatology nurse wherein a detailed review of a subject’s medical history, a special assessment of cirrhosis severity and cirrhosis related events or symptoms (including animal naming test [ANT] [23]) as well as previous and current pharmacological treatments is performed. Information on demographics and medical history is collected from the patient and from medical records.
- A detailed clinical examination including blood pressure, weight, height, waist and hip circumference, and assessment of relevant lifestyle factors.
- Questionnaires to assess disease burden and health-related quality of life (HRQoL).
- Comprehensive blood panels including markers for blood, electrolyte, liver, and lipid status as well as blood samples for biobanking.
- Vibration-controlled transient elastography (VCTE) and controlled attenuation parameter (CAP) with Fibroscan™.
- Tests of muscle strength and physical performance, including the Short Physical Performance Battery (SPPB), hand-grip strength (HGS), and Liver Frailty Index (LFI).
- Estimated body composition measured by bioelectrical impedance measurement according to clinical routine (only at University Hospital in Linköping).
- 20 min MR examination including BCP with MAsS, portal flow, MRE, and HCC-screening.

### Follow-up

After inclusion and collection of baseline data, follow-up visits are performed after 6, 12, 18 and 24 months (Table 3). The study includes 5 visits (4 MR-examinations and a final follow-up visit) with 6 months interval if no liver lesions are found on MR-examination. If a nodule is defined as LI-RADS > 3, the patient has reached a significant endpoint and no further study visits will be performed (follow-up data will be collected through chart review). If a LI-RADS-3 is found on MR-examination, the time interval between subsequent MR-examinations is shortened from every 6 months to every 4 months during a 12-month period. This adds an extra MR-examination for patients with LI-RADS-3 (cumulative total of 5 MR-examinations instead of 4 during the study period) and therefore the cumulative number of visits is also increased from 5 to 6 visits (Fig. 1).

**Table 3:** Flow chart of inclusion and follow-up visits

Procedure/data collection	Baseline (visit 1)	Follow-up (visit 2–4)	End of study (visit 5)
**Evaluation incl/excl criteria**	X		
**Informed consent**	X		
**Demography**	X		
**Medical history**	X		
**Family history**	X		
**New diagnoses**		X	X
**Health changes**		X	X
**Life-style factors**	X	X	x
**Concomitant medications**	X	X	X
**Clinical work-up**	X	X	X
**HE-assessment (ANT)**	X	X	X
**QoL questionnaires**	X	X	X
**Muscle function test**	X	X	X
**FibroScan**	X	X	X
**Blood panels**	X	X	
**Blood samples for research**	X	X	
**MR-examination**	X	X	

After end of the study period, all participants are included in regular follow-ups and HCC-screening according to clinical praxis (i.e.*,* ultrasonography every 6 months).

### Questionnaires

Patients will undergo validated questionnaires to assess quality of life and validated physical functional tests relevant to cirrhosis:

I. Patient reported quality of life
  EQ-5D-5L: This questionnaire was developed by the EuroQol Group in 2009 as a measure of health-related quality of life. The descriptive system comprises five dimensions: mobility, self-care, usual activities, pain/discomfort, and anxiety/depression [24].
  CLDQ: The chronic liver disease questionnaire (CLDQ) is a self-reported questionnaire which has undergone repeated validation [25, 26].
  SHS: The short health scale is a validated self-administered visual analog scale questionnaire studied in both inflammatory bowel disease and irritable bowel syndrome [27–29].
II. Muscle function and physical frailty tests
  SPPB and hand grip strength: Short Physical Performance Battery (SPPB) [30] and hand grip strength [31, 32] were originally developed and validated in elderly populations. However, they have shown to predict mortality in both elder and younger patients with severe liver disease [33].
  Hand-grip strength
  Liver frailty index has been developed as an instrument to screen for frailty in patients with cirrhosis.

### Blood panels

**Fig. 2:**
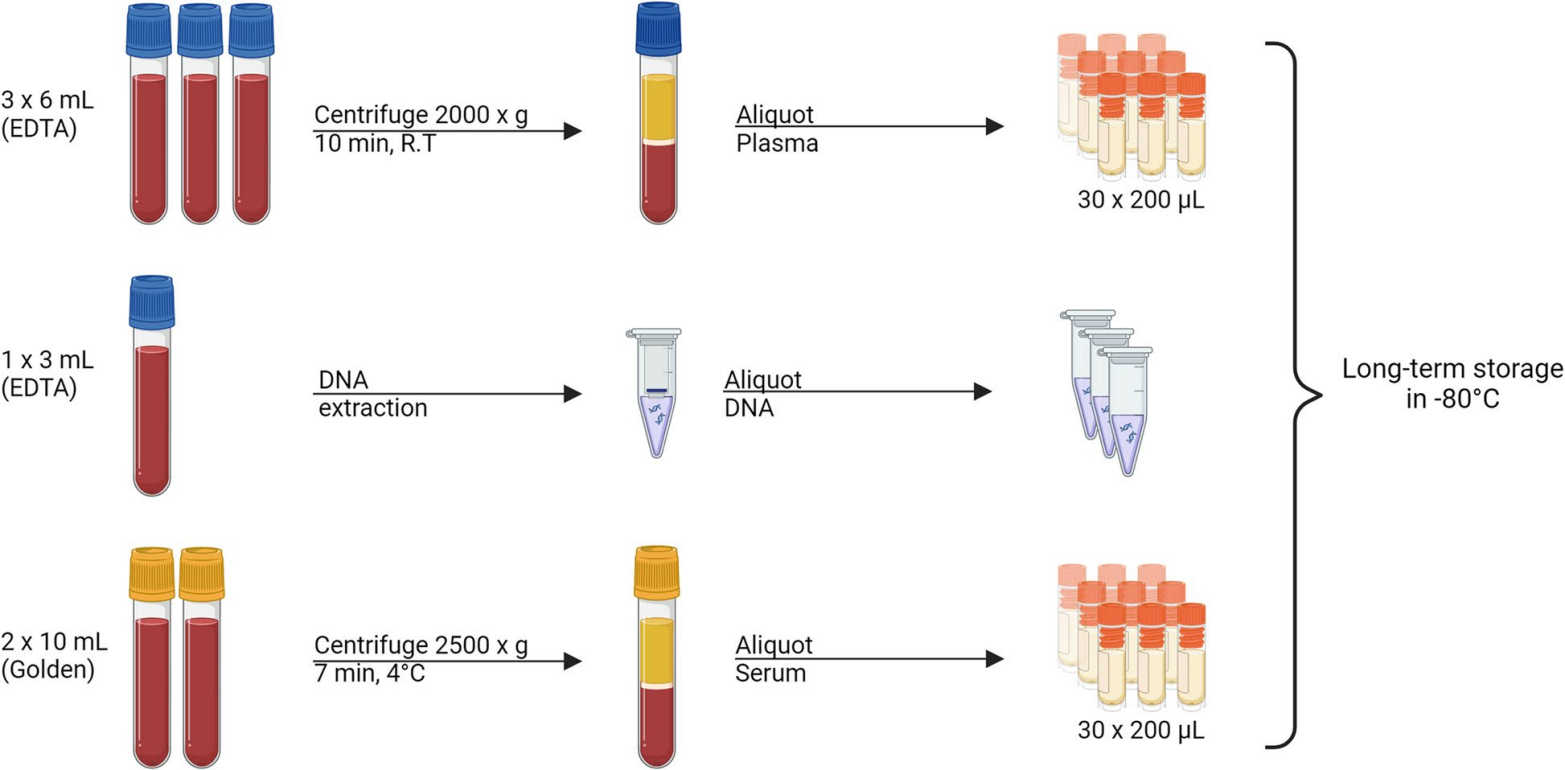
Overview of blood sample acquisition and biorepository. Created with BioRender.com

Fasting blood samples will be collected from participants after an overnight fast. Samples will be stored at the Linköping Biobank Facility. The biobank facility is a state-of-the-art facility for quality-controlled storage in secure freezers. Collected samples are outlined in Fig. 2, from all participants blood, serum and plasma will be collected.

All subjects consent so that samples can be transferred within and outside the EU/EEA and that samples can be sent for analyses to private companies in the biotechnology and/or pharmaceutical industry.

### Clinical hematology/biochemistry/immunology/virology

Several blood, serum and plasma markers will be analyzed (Table 4) to obtain a biochemical profile and to calculate previously identified biomarker algorithms associated with prognosis in cirrhosis (i.e.*,* MELD, CPT).

**Table 4:** Detailed blood/serum/plasma work-up

Blood/Serum/Plasma markers	
**Complete blood count**	
• Hemoglobin, hematocrit, mean corpuscular value, mean corpuscular hemoglobin, mean corpuscular hemoglobin concentration, platelets, white blood cells.	
**Liver tests**	
• Albumin, bilirubin, ALT, AST, ALP, γGT, PT (INR), ammonia, high sensitive C-reactive protein	
**Iron studies**	
• Iron, transferrin saturation, total iron binding capacity, ferritin	
**Minor kidney function panel**	
• Sodium (Na), potassium (K), creatinine	
**Metabolic tests**	
• Cholesterol, triglycerides, LDL, HDL. fasting glucose, insulin, apolipoprotein AI and B	
**Direct alcohol marker**	
• Phosphatidylethanol	

*Abbreviations:* *ALP* alkaline phosphatase, *ALT* alanine aminotransferase, *ANA* antinuclear antibody, *AST* aspartate aminotransferase, *γGT* gamma-glutamyl transferase, *INR* international normalized ratio, *PT* prothrombin time

### Vibration-controlled transient elastography

Transient elastography (TE) relies on a transient mechanical vibration used to induce a shear wave in a tissue. The propagation of the shear wave is then tracked using ultrasound to assess the shear wave speed. A specific implementation of 1D-TE, vibration-controlled TE (VCTE), has been developed to assess the average liver stiffness that correlates with liver fibrosis assessed by liver biopsy [33]. However, several studies have shown liver elasticity to also correlate with the risk of liver-related morbidity and mortality in patients with, and without, liver cirrhosis. In this study, it will be implemented using FibroScan®, including M- and XL-probe as well as Controlled attenuation parameter (CAP). CAP measures liver ultrasonic attenuation on the signals acquired by the FibroScan®. Principles of CAP measurements has been described elsewhere [34].

### Magnetic resonance imaging

MRI data acquisition is performed using a 3 T MRI scanners, Philips Ingenia (Philips Healthcare, Best, The Netherlands) at the Center for Medical Imaging and Visualization (CMIV), Linköping University Hospital and 3 T MR-scanners, Siemens Vida (Siemens, Erlangen, Germany) at the County Hospital in Jönköping and the District Hospital in Eksjö. We have devised a multimodal MR-protocol for this project that includes a range of specific MR-techniques (Table 5). In short, the protocol includes determination BCP with MAsS, MRE, and HCC screening.

**Table 5:** Overview of the 20-min MRI examination. The sequences are listed in the scan order and highlighted in bold text. The imaging markers stemming from the individual sequences are listed in the last row. The body-composition profiling sequences (BCP) differ depending on abdominal or thigh coverage; in the abdominal sequences visceral adipose tissue (VAT) and abdominal subcutaneous adipose tissue (BCP) volumes as well as spinal erectors fa-free muscle volume (FFMV) and muscle fat infiltration (MFI) are measured. In the thigh sequences thigh FFMV and MFI are measured. The liver proton density fat fraction (PDFF) is measured using the mDixon quant sequence. The T2* liver sequence can be used to measure liver iron content (LIC). The dynamic Dixon sequences taken at 0,45,70,180 after bolus injection of gadoterate meglumine (Dotarem®) can measure signal intensity (SI) contrast based hepatobiliary markers. Lastly the two MRE sequences can measure the liver stiffness (|G*|)

Sequence Name	BCP-Abdomen	BCP-Thigh	mDixon Quant	T2* Liver	mDixon Dynamic	MRESE	MREGRE
Mode	T1-weighted	T1-weighted	Quantitative Imaging	T2-weighted	Dynamic Imaging (0/45/70/180 s)	MR Elastography	MR Elastography
TR/TE (ms)	“shortest”/1 (3.4/0.98)	“shortest”/"shortest” (3.5/1.13)	“shortest”/"shortest” (6.9/0.96)	“shortest”/2.30 (8.2/2.3)	“shortest”/"shortest” (3.2/1.14)	1000/53.84	50/"shortest” (50/20)
Echo-train length	2	2	6	N/A	2	1	1
Flip angle (°)	10	10	3	10	10	90	30
no. slices	37	71	77	37	131	4	4
FOV RL/AP/FH (mm)	530/371/222	540/368/284	400/350/231	530/371/222	380/312.14/229.25	370/314.5/43	450/403.1/43
ACQ voxel size RL/AP/FH (mm)	3.3 × 3.6 × 6	2 × 2.2 × 4	3 × 2.99 × 3	2.20 × 3.59 × 6	1.75 × 1.75 × 3.5	4.67 × 4.67	1.5 × 4.67
ACQ matrix size	160 × 104	264 × 167	132 × 118	240 × 102	216 × 178	80 × 66	300 × 86
Reconstruction matrix	160	288	192	240	448	320	384
NSA		1	1	1	1	1	1
Breath hold	“yes”	“no”	“yes”	“yes”	“yes”	“yes”	“yes”
Compressed SENSE	“no”	“no”	“no”	“no”	“yes” (4)	“no”	“no”
Imaging marker(s)	VAT, ASAT, liver volume, spleen volume, spinal erectors, FFMV and MFI	Thigh FFMV and MFI	PDFF	LIC	SI	(|G*|)	(|G*|)

**Fig. 3:**
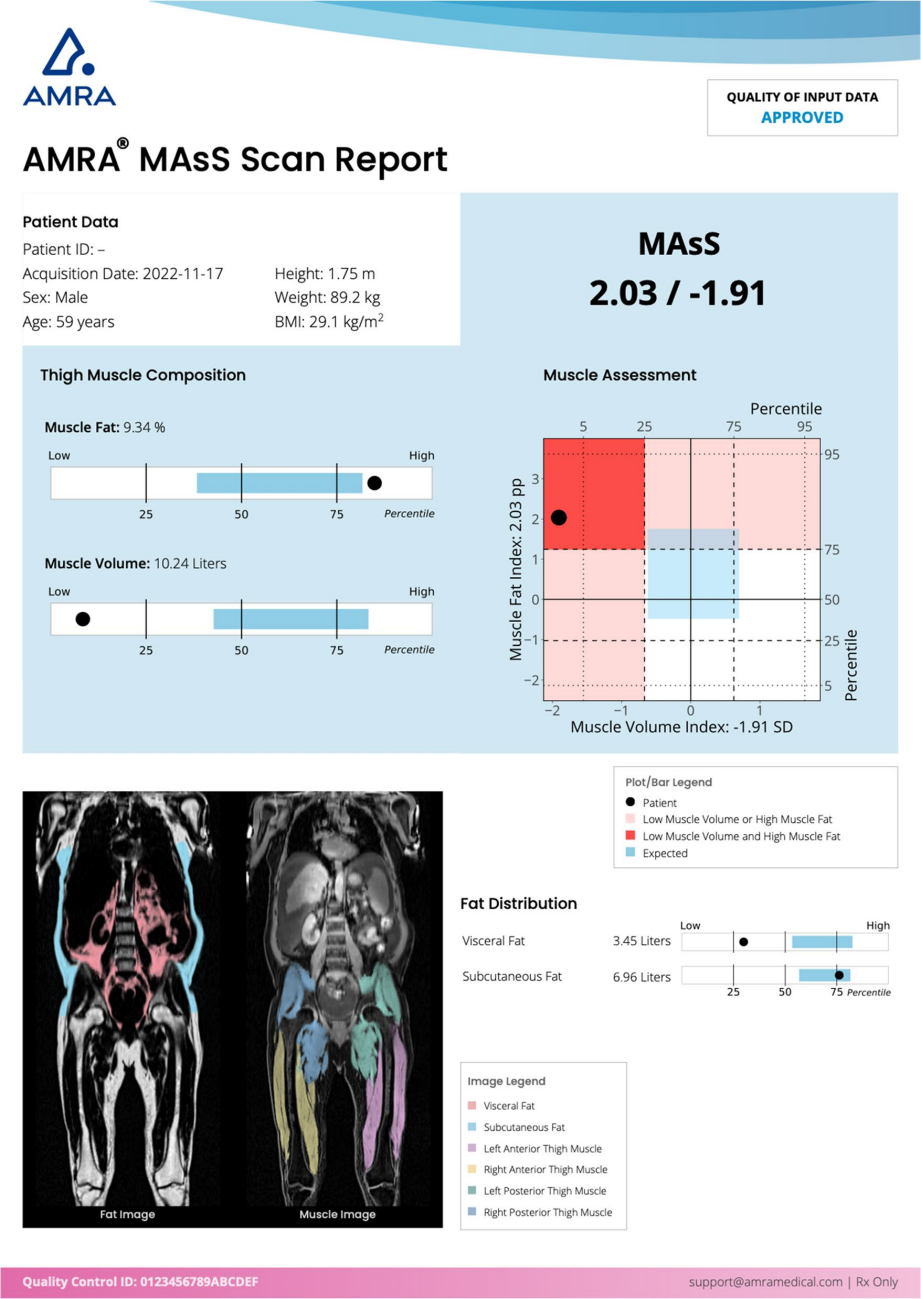
An example of clinical report for body composition profiling (AMRA® Profiler 4 MAsS Scan), including whole-body water-fat separated imaging examples (bottom left), the muscle assessment score (MAsS; blue region of the report) which are the biomarkers used in the primary aim to assess sarcopenia, as well as measurements of abdominal adiposity (bottom right)

The body composition includes thigh fat-free muscle volume (FFMV) and muscle volume z-sore (FFMVz), thigh muscle fat infiltration (MFI) and sex-adjusted MFI, liver fat content (PDFF), visceral adipose tissue volume (VAT), abdominal subcutaneous adipose tissue volume (ASAT), spleen volume, spinal erector FFMV and MFI as well a skeletal muscle index at the 3rd lumbar vertebrae (L3-SMI; Fig. 3) measured by AMRA Medical AB (Linköping, Sweden) [21, 35–38]. 2D MRE (Resoundant, USA) is acquired as shown in Table 5 and processed as per the QIBA guideline [39]. HCC screening by aMRI will be conducted using standard gadolinium contrast agent (Dotarem®) and radiological reading.

The MR-protocol is efficiently condensed, and the MR data will be acquired within c. 20 min.

### Time plan and implementation

The project involves patient recruitment, data acquisition, and data analysis. Patient recruitment started in 2021. The goal is to complete patient inclusion in 2023 (ending the study with the last patients’ last visit in 2025).

## Discussion

This prospective study, on highly phenotyped cirrhosis patients, aims to make a unique and comprehensive assessment on the natural history of cirrhosis. By utilizing sequential, same-day examinations over a period of 2 years, this study aims to evaluate non-invasive biomarkers based on a clinically viable 20 min MRI scan in search of prognostic factors associated with ESLD, HCC, sarcopenia and liver-related morbidity and mortality.

Chronic liver diseases affect a large proportion of individuals globally. Albeit the progression rate among etiologies differs, all individuals with a chronic liver disease have an increased risk of developing cirrhosis and end-stage-liver disease complications, including HCC and decompensation (i.e.*,* ascites, bleeding esophageal varices, jaundice, liver encephalopathy and hepatorenal syndrome).

Cirrhosis is recognized as the 11th most common cause of death when including HCC, accounting for 3.5% of all deaths worldwide [2]. The estimated prevalence of cirrhosis is currently 0.1 and 0.27% in Europe and the US, respectively, and these numbers are expected to rise due to the obesity epidemic leading to increased numbers of patients with NAFLD. The worldwide prevalence of NALFD is approximately 20–30% and about 5–10% of these patients will develop an advanced form of liver disease in the future, which entails advanced fibrosis, cirrhosis or ESLD [40–42].

Despite portal hypertension being the main pathophysiological cause of decompensation in cirrhosis, the assessment of portal hypertension has historically been reactive and oriented towards clinical observation for apparent symptoms or signs of decompensation. First after these clinical signs appear the pharmacological treatments, invasive interventions, or surgical actions are initiated. However, in studies on asymptomatic patients with cirrhosis without varices, portal hypertension evaluated by increased HVPG has been observed to predict future clinical decompensation as well as HCC [43]. Furthermore, a meta-analysis of studies on treatment with unselective beta-blockers for portal hypertension in patients with compensated cirrhosis found that prophylactic treatment lowered the risk of future decompensation [44]. Hence, early treatment of portal hypertension is an important factor for patient outcome, and therefore there is a desire for physicians to be able to predict future decompensating events by non-invasive methods to initiate treatments that prolong survival. Thus, we expect the optimal way forward in clinical care for cirrhosis and portal hypertension lies in developing solutions to become more proactive in identifying and treating early portal hypertension. By developing robust, prognostic biomarkers, we will have the opportunity to move the field of hepatology into a proactive era and hopefully use interventions for tailored ‘*treat to target’* interventions.

Hepatocellular carcinoma is the fourth leading cause of cancer-related deaths worldwide, with an estimated annual incidence of 2–5% per year in patients with liver cirrhosis (albeit the risk differs by underlying etiology) [45, 46]. The World Health Organization is estimating that more than 1 million patients will die from HCC in 2030 [45]. Since curative treatment options are available for HCC if diagnosed early, surveillance should be initiated in all patients with cirrhosis including biannual investigation with an accurate imaging modality, most commonly ultrasonography. However, in the presence of an HCC-suspected lesion, adding a second imaging modality (i.e.*,* MRI or computed tomography) is recommended. Albeit ultrasonography is the most common modality for HCC surveillance, some downsides need to be recognized. Recent studies have found MRI to have a higher HCC detection rate compared to ultrasound [9, 47]. Furthermore, a high frequency of patients with cirrhosis also have obesity and visualisation limitations with ultrasound is not uncommon, in which cases MRI is recommended [48]. Magnetic resonance imaging has previously been limited by high equipment costs and relatively long examination times, but an aMRI protocol has recently been shown to have a high sensitivity and specificity for HCC irrespective of protocol or addition of contrast agent [10, 49, 50].

Sarcopenia is recognized to be an important predictor of mortality in cirrhosis and HCC [51, 52]. The components of the MAsS have in recent publications been shown to assess sarcopenia invariant to common confounding factors such as age and body habitus [15] – a common flaw in proposed sarcopenia definitions and thus a potential issue in for instance NAFLD with its link to the obesity epidemic.

MAsS, also applied in this project, has been shown to be able to be an independent predictor of all-cause mortality and identifies vulnerable patients with fatty liver disease [16, 17].

Therefore, in this study recruited patients with cirrhosis will undergo HCC-screening, utilising MRI instead of ultrasonography, biannually, for 2 years – investigating both the presence of HCC and biomarkers associated with ESLD, such as sarcopenia and associated morbidities. Methodologically, we will assess sarcopenia, clinically significant portal hypertension, liver stiffness and HCC with a single short MR-examination. Sarcopenia will be assessed using BCP with MAsS. The BCP analysis is acquired with a 8 min neck-to-knee MR-examination which will also provide measurements of abdominal fat compartments and spleen volume [21, 53]. We will also measure L3-SMI which previously has been used on computed tomography for measuring sarcopenia and compare the predictive performance against MAsS. Liver stiffness will be assessed by Fibroscan™ and standard clinical MRE methods. HCC screening will be performed using so-called abbreviated MRI (aMRI).

A major strength of our study is that included patients undergo same-day examinations allowing for accurate comparisons between the results of different tests and modalities utilized. Another strength is that it is a longitudinal study repeating the same investigations over a 2-year period as well as an extra visit after the last scheduled visit for all patients who experience decompensation. This allows us to follow the natural history of cirrhosis and investigate how imaging and other biomarkers relate to actual clinical outcomes i.e.*,* decompensation, HCC, and sarcopenia.

During an era where non-invasive test (NIT) proves to be non-inferior or even superior to invasive, diagnostic interventions often entitled as gold standard, the need for implementing, validating, and further evolving available non-invasive biomarkers in prospective cohorts are crucial. Albeit VCTE and NITs have revolutionized the field of hepatology – their strength mainly lies in their high negative predictive values. However, albeit, VCTE has recently shown to match the prognostic capability as histological fibrosis stage, it has some limitations – the main one being its inability to also screen for HCC.

Furthermore, few biomarkers have been validated for their responsiveness to change over time and their correlation with disease activity. Utilising magnetic resonance imaging together, and in comparison, with readily available biomarkers we strive to improve care for patients with liver cirrhosis through development of diagnostic, prognostic, and responsive blood-based and imaging biomarkers to sharpen personalized medicine in the field hepatology.

## Data Availability

Data can be accessed upon request but will not be openly available. Point of contact: Mattias Ekstedt, mattias.ekstedt@liu.se.

## Abbreviations

aMRI: Abbreviated MRI
BCP: Body composition profile
CAP: Controlled attenuation parameter
CLDQ: Chronic liver disease questionnaire
CSPH: Clinically significant portal hypertension
CTP: Child-Turcotte-Pugh
ESLD: End-stage liver disease
GDPR: General Data Protection Regulation
FFMV: Fat free muscle volume
HCC: Hepatocellular carcinoma
HRQoL: Health related QoL
HVPG: Hepatic venous pressure gradient
ICH-GCP: International Council for Harmonisation of Technical Requirements for Pharmaceuticals for Human Use – Good Clinical Practice
LI-RADS: Liver imaging reporting and data system
MELD: Model for end-stage liver disease
MFI: Muscle fat infiltration
MR: Magnetic resonance
MRE: MR elastography
MRI: MR imaging
NAFLD: Non-alcoholic fatty liver disease
PDFF: Proton density fat fraction
SHS: Short health scale
SPPB: Short physical performance battery
TE: Transient elastography
QoL: Quality of life
SAT: Subcutaneous adipose tissue
VCTE: Vibration controlled TE
VAT: Visceral adipose tissue.
